# Supplementary material for: TFAM’s Contributions to mtDNA Replication and OXPHOS Biogenesis Are Genetically Separable
Source: Cells. 2022 Nov 24;11(23):3754. doi: 10.3390/cells11233754 (PMC9739059; doi:10.3390/cells11233754)
Supplement: Supplementary file 1 [file cells-11-03754-s001.zip › Figure S1.pdf]

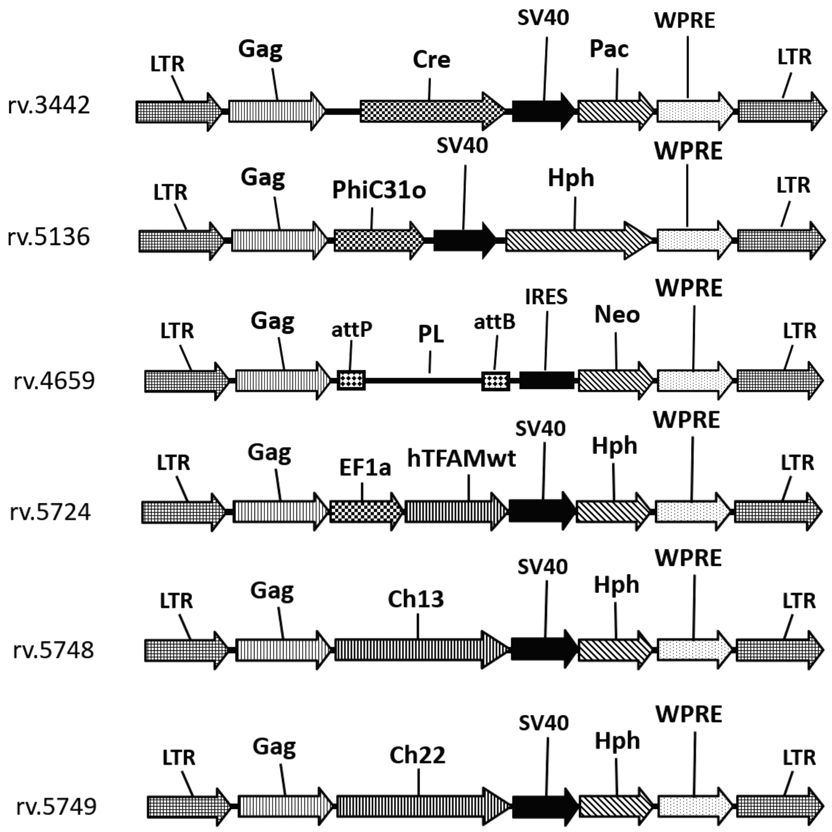

Figure S1. Maps of vectors used in this study. Designations: attP, attB, recombination sites for PhiC31 recombinase; Cre, gene encoding the bacteriophage P1 Cre recombinase; EF1a, CpG-free EF1α promoter; Gag, truncated retroviral Gag protein; Hph, hygromycin phosphotransferase, hygromycin resistance gene; hTFAMwt, wild-type hTFAM; IRES, internal ribosome entry site; LTR, retroviral long terminal repeat; Neo, G418 and kanamycin resistance gene; Pac, puromycin resistance gene; PhiC31o, optimized PhiC31 recombinase gene; PL, polylinker; SV40, promoter of the SV40 virus; WPRE, woodchuck hepatitis virus posttranscriptional regulatory element.

The utility of the vectors is as follows:

rv.3442 (Addgene#184852). A retroviral vector encoding Cre recombinase and puromycin resistance. Used to deliver Cre recombinase to effect wt hTFAM excision in 143B#6 cells.

rv.5136 (Addgene#184853). A retroviral vector encoding PhiC31 recombinase and hygromycin resistance. Used to deliver PhiC31o recombinase to effect TFAMvar excision.

rv.4659 (Addgene#184854). A general-purpose retroviral vector encoding G418 resistance. Contains a polylinker flanked by attP and attB sites for PhiC31 recombinase. Used to generate retroviral constructs encoding PhiC31-excisable chTFAM variants.

rv.5724. A retroviral vector encoding CpG-free EF1α promoter-driven wt hTFAM and hygromycin resistance. Used for Ch13 and Ch22 complementation.

rv.5748. A retroviral vector encoding Ch13 and hygromycin resistance. Used for Ch13 and Ch22 complementation.

rv.5749. A retroviral vector encoding Ch22 and hygromycin resistance. Used for Ch13 and Ch22 complementation.
