# Supplementary material for: TFAM’s Contributions to mtDNA Replication and OXPHOS Biogenesis Are Genetically Separable
Source: Cells. 2022 Nov 24;11(23):3754. doi: 10.3390/cells11233754 (PMC9739059; doi:10.3390/cells11233754)
Supplement: Supplementary file 1 [file cells-11-03754-s001.zip › Figure S2.pdf]

	hTFAM DNA contacts			chTFAM equivalent positions
	At LSP	At HSP1	At Nonspecific DNA	
HMG1	K51			K46
	K52	K52	K52	R47
	S55		S55	S50
	S56			A51
	Y57	Y57	Y57	Y52
	L58	L58		F53
	S61…		S61	L56
		L65	L65	Q60
			K69	R64
	T77	T77		S72
	T78	T78	T78	L73
	I81	I81	I81	V76
	R82		R82	K77
	W88		W88	W83
	R89	R89	R89	R84
	Q100		Q100	E95
	Y103	103		R98
Linker	H137		H137	L132
			K139	K134
	R140	R140	R140	R135
			M143	F138
		K146		K141
	K147			R142
	T150…			T145
HMG2			K156	K151
	R157	R157	R157	R152
	R159		R159	R154
	Y162	Y162	Y162	F157
	N163	N163	N163	N158
		A167		S162
	P178	P178	P178	P173
	Q179	Q179		T174
		K186	K186	F181
	W189	W189	W189	W184
			E208	K203
	Y211			Y206
C-Ter	R232			R227
	R233			S228
	T234			R229

Figure S2. Amino acid residues in contact with DNA in hTFAM and corresponding residues in chTFAM.
