# Supplementary material for: TFAM’s Contributions to mtDNA Replication and OXPHOS Biogenesis Are Genetically Separable
Source: Cells. 2022 Nov 24;11(23):3754. doi: 10.3390/cells11233754 (PMC9739059; doi:10.3390/cells11233754)
Supplement: Supplementary file 1 [file cells-11-03754-s001.zip › Table S2 oligos.pdf]

Table S2. Oligonucleotides used in this study.

Purpose	Name	Sequence	Amplicon, bp
**Genotyping**			
Genotyping of the ρ^0 state	hMitF	AATGTCTGCACAGCCACTTTCCAC	Mit=901
	hMitR	TCGTAGTGTTCTGGCGAGCAGTTT	
	hPOLRMTf	AGAATGTGGCAGTGGAGATG	POLRMT=371
	hPOLRMTr	GCGAGTGCTGCCCCGAC	
	H18Sf	CGGACAGGATTGACAGATTGA	18S=389
	H18Sr	AGCTTATGACCCGCACTTAC	
Genotyping of hTFAM excision in 143B#6 cells	ExF2	CGCCTCAATCCTCCCTTTATC	WT=408 Excised=251
	ExR2	CACACCTGGTTGCTGACTAA	
	ExInt2	GCATCTGGGTTCTGAGCTTTA	
Genotyping chTFAM	ChickF	CCAGAAGCAAGTGTACGAAGAG	379
	ChickR	CGCCTCCCAGGATTTCATTT	
Genotyping PhiC31 recombinase	PhiC31F1	AAGCGCCAACGAGGATAAG	308
	PhiC31R1	TCACGTTGCCCTGTCTAAAG	
Genotyping chTFAM variant excision	F	ACCTACCCGAGTCGGACTTT	WT=1264/1279
	R1	GTTATTGCTTGGGATGTACTTGG	Ex=370
**RT-qPCR of transcripts**			
hHPRT	hHPRTf	CGAGATGTGATGAAGGAGATGG	N/A
	hHPRTr	TTGATGTAATCCAGCAGGTCAG	
hMT-ND1	hND1F	GAAGTCACCCTAGCCATCATTC	N/A
	hND1R	GCAGGAGTAATCAGAGGTGTTC	
hMT-ND6	hND6F	CCACACCGCTAACAATCAATAC	N/A
	hND6R	GTTTCTGTTGAGTGTGGGTTTAG	
hMT-RNR2 (16S)	h16Sf	GAAACCAGACGAGCTACCTAAG	N/A
	h16Sr	GGTTTGTCGCCTCTACCTATAAA	
hMT-CO1	hCox1F	CTAGCAGGTGTCTCCTCTATCT	N/A
	hCox1R	GGCGTTTGGTATTGGGTTATG	
hMT-CO2	hCox2F	CTCAGACGCTCAGGAAATAGAA	N/A
	hCox2R	TCGTTGACCTCGTCTGTTATG	

mtCN determination by dddPCR

mtDNA copy number determination by ddPCR	NucF	AACTTGTAAGTGGTAGTGCATAGA	N/A
	NucR	GTAGGAGGACATTTGAGGAGTG	
	NucProbe	FAM-TCAGGCAGACTGACACTAGAGTTCACA-BHQ1	
	MitF	CTGATCAGGGTGAGCATCAAA	
	MitR	GAATGATGGCTAGGGTGACTTC	
	MitProbe	Hex-TGCGAGCAGTAGCCCAAACAATCT-BHQ1	
